# Esophageal Lichen Planus: The Efficacy and Safety of Tacrolimus in Reducing Inflammation and Need for Dilation

**DOI:** 10.14309/ctg.0000000000000752

**Published:** 2024-07-15

**Authors:** Keshav Kukreja, Ambuj Kumar, Charles Camisa, John Jacobs, Joel E. Richter

**Affiliations:** 1 Division of Digestive Diseases and Nutrition, Joy McCann Culverhouse Center for Swallowing Disorders, University of South Florida Morsani College of Medicine, Tampa, Florida, USA;; 2 Department of Dermatology and Cutaneous Surgery, University of South Florida Morsani College of Medicine, Tampa, Florida, USA.; 3 Research Methodology and Biostatistics Core, Office of Research, University of South Florida Health, Tampa, Florida, USA

**Keywords:** esophagitis, lichen planus, dysphagia, tacrolimus

## Abstract

**INTRODUCTION:**

Esophageal lichen planus (ELP) is a rare inflammatory disease most seen in middle-aged White women, manifested by sloughing mucosa, thick exudate, and proximal strictures. Most case reports and small series highlight using steroids and other immunosuppressants. To the best of our knowledge, oral tablet tacrolimus has not been studied. We aimed to assess the change in ELP after oral tacrolimus treatment.

**METHODS:**

The primary outcome was the efficacy of tacrolimus objectively through our scoring system, ELP Severity Score (ELPSS). All consecutive adults with ELP who underwent more than one esophagogastroduodenoscopy by 2 esophagologists and being treated with tacrolimus or other treatment were eligible for inclusion in this retrospective cohort study. Inflammation and fibrostenotic disease were graded using the novel ELPSS.

**RESULTS:**

Twenty-two patients met the inclusion criteria. Half (11) received tacrolimus (dose 1–2 mg twice daily), and half (11) received other therapy (i.e., cyclosporine, topical steroids, or none). Mean ELPSS on the first esophagogastroduodenoscopy, extraesophageal manifestations of disease, presenting symptoms, and baseline characteristics were similar between groups. Among patients on Tac vs No-Tac, there was a statistically significant improvement in ELPSS (mean difference 1.8 pts; 95% confidence interval 0.25–3.38; *P* = 0.02). Response rate was 89% with Tac vs 30% with No-Tac (*P* = 0.025). All 22 patients underwent bougie dilation safely with a mean diameter of 16 mm achieved. Patients on Tac also required less frequent dilation.

**DISCUSSION:**

Oral tablet tacrolimus reduced the inflammatory and fibrostenotic components of ELP. Thus, low-dose oral tacrolimus is safe and should be considered in patients with more severe disease.

## INTRODUCTION

Lichen planus (LP) is a common mucocutaneous disorder usually involving the skin and oral mucosa. It can also involve the anogenital area, hair, nails, and esophagus. The prevalence varies widely and has been reported to affect 0.2%–5% of the population (1,2). Esophageal lichen planus (ELP) is a less commonly encountered or recognized entity. In literature, approximately 200 cases have been described, with the first 2 cases reported in 1982 (3). Evidence from research suggests that esophageal manifestations can be evident in up to 50% of patients with LP and even the presenting sign of LP in up to 48% of patients (4).

Most patients with ELP are White, and there is a strong female gender predilection (5). Typical presenting symptoms include dysphagia, odynophagia, weight loss, or heartburn refractory to traditional therapies. Endoscopic findings of ELP include predominantly upper esophageal strictures, and inflammation, which is manifested by pale, edematous mucosa, varying degrees of thick white exudate, and easily sloughing mucosa. The histopathology, when consistent, can be reassuring in making the diagnosis. Often, nonspecific findings are described including (i) a dense band-like subepithelial or junctional lymphocytic infiltrate, (ii) luminal fibrinoinflammatory exudate and granulation tissue, (iii) degeneration of the basal layer, and (iv) vacuolation and degeneration causing necrosis of keratinocytes, termed “Civatte bodies” (6).

Given the rarity of ELP, currently, there is no consensus or guidelines on optimal medical management. The treatment of ELP follows a similar mantra to other inflammatory disorders of the esophagus, such as eosinophilic esophagitis (EoE) (6). The aim is to treat inflammation and fibrosis simultaneously with medications and esophageal dilatation, respectively.

Most case reports and small series highlight the use of steroids (7–13). Albeit rapidly effective as an anti-inflammatory agent, their long-term use has significant side effects, and patients often have recurrence of symptoms with doses less than 10 mg/d. The use of topical steroids in ELP was likely inspired by their efficacy in EoE. Both current formulations, fluticasone and budesonide, have been useful for ELP, showing up to a 67% success rate, usually in milder forms of the disease (7,8,10,11,13–19).

Numerous other therapies, such as retinoids, adrenocorticotropic hormone, cyclosporine, intralesional steroids, and rituximab, have been reported (6). Unfortunately, a majority of the aforementioned therapies were from small case series with inconsistent efficacy because of side effects and widely varying follow-up time.

Topical tacrolimus has been studied extensively for oral LP with strong results in achieving clinical and histologic remission in comparison with other therapies. The rationale of utilizing this medication is its mechanism of action, which involves reducing T-cell proliferation. The topical form of this medication for ELP has been reported in conjunction with steroids only (20,21). The oral tablet form of tacrolimus, however, has not been reported previously as a therapy for ELP. Working alongside a dermatologist with expertise in inflammatory and autoimmune conditions, we have been using tablet tacrolimus in ELP patients for nearly 10 years.

## METHODS

### Patient selection and data collection

We performed a retrospective cohort study. All consecutive adult patients with a confirmed diagnosis of LP who underwent esophagogastroduodenoscopy (EGD) at the University of South Florida and Joy McCann Culverhouse Center for Swallowing Disorders between January 1, 2011, and July 1, 2020, were eligible for inclusion. All included patients were seen in the clinic and had EGD performed by 1 of 2 esophagologists. Any patient younger than 18 years or not treated at University of South Florida and Joy McCann Culverhouse Center for Swallowing Disorders was excluded. All data were extracted from the electronic medical records. Patients were identified by cross-referencing the *International Classification of Diseases* code for LP (L43.8) with EGD (Z13.810 or Current Procedural Terminology codes 43235–43259). Approval for this study was obtained from the Institutional Review Board at the University of South Florida.

We collected the following data: age, gender, weight, height, body mass index (BMI), social history (tobacco and alcohol), medical history (hepatitis C or B infection), medication history (those associated with oral lichenoid reactions), symptomatology, and any ELP treatment(s) before and during presentation to our institution. When available, history of endoscopic intervention (dilation) and previous medication treatments were recorded.

One cohort was treated primarily with tacrolimus and one with other therapies (cyclosporine, steroids, etc). All patients had upper endoscopy, biopsy, and bougie dilatation (Maloney or Savary) performed at baseline and as needed based on recurrence and severity of dysphagia. Data on the endoscopic procedure and postprocedural AEs were extracted from the endoscopic database and electronic medical record, respectively. Procedural data for ELP included the presence of mucosal abnormalities (pale/edema, exudate, and sloughing) and strictures (total number, severity, location, and dimensions). In addition, we recorded the type of esophageal dilation performed (Savary or Maloney), largest diameter bougie passed, and any other cointerventions (e.g., medication use). Data regarding postprocedural AEs, including any treatment needed (including repeat endoscopic interventions performed because of AEs), were also recorded.

Data regarding the time to repeat dilation were recorded. Follow-up duration was recorded from time of first EGD to date of last follow-up or death.

### Objectives

**Figure 1.**
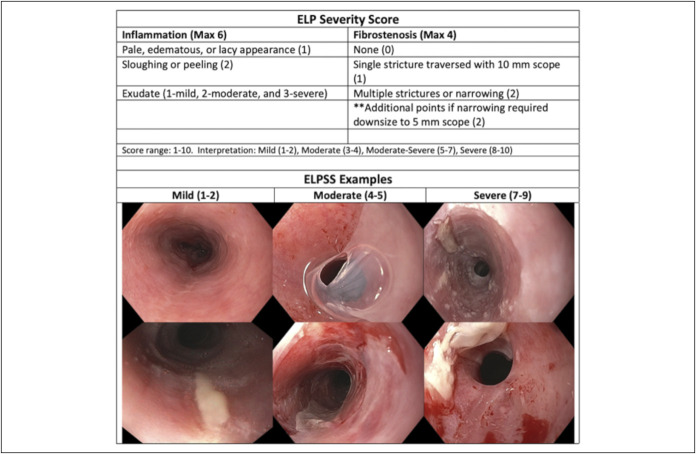
Illustration of the mild (left), moderate (middle), and severe (right) ELPSS. In the inflammatory component, sloughing or peeling occurs spontaneously or with the passage of scope only—not after dilatation. Exudate classification: mild—scattered punctate exudates; moderate—short serpiginous plaques; and severe—long, thick, serpiginous plaques. Within the fibrostenosis component, “multiple narrowing” describes more than one stricture or general narrowing involving more than one location in the esophagus. Adapted from ref. (6). ELP, esophageal lichen planus; ELPSS, Esophageal Lichen Planus Severity Score.

The primary objective was assessing the efficacy of tacrolimus as a treatment for ELP, which we accomplished by developing a scoring system to gauge disease severity (Figure 1). Secondary objectives were assessing the safety of tacrolimus and dilatation and any associated complications including bleeding, perforation, or pain.

### Grading disease severity and prescribing tacrolimus

The decision to prescribe tacrolimus was made by a senior dermatologist with experience in bullous and autoimmune skin diseases. Tarcolimus was used when other more traditional drugs had failed. The number of previous medications varied from none (patients we made diagnosis of LP) to 3. The same dermatologist assessed the severity of the LP on oral examination, reviewed all endoscopic findings, by history was aware of other sites of LP and contraindications to the drug and especially in our early experience.

Inflammation and fibrostenotic disease were graded using the ELP Severity Score (ELPSS), which we developed using the EoE Reference Score as a model. The ELPSS encompasses the most common findings we saw in ELP patients and was devised after review of 108 high-quality endoscopic reports. Within the inflammatory component, patients got 1 point for abnormal mucosa that was either pale, edematous, or lacy; 2 points for spontaneous peeling; and 1–3 points for exudate based on severity. Fibrostenotic component was graded with 1 point for a single stricture OR 2 points for multiple strictures or narrowing. Patients also received 2 additional points if traversing the stenosis required a 5-mm XP scope, indicating very severe stenosis (Figure 1). We found there to be minimal inter-rater variability among 3 blinded raters who reviewed 42 endoscopic reports independently (Table 1). The Lin's Concordance Correlation coefficients comparing their total ELPSS for the baseline EGD were 0.77 (raters 1 and 2), 0.74 (raters 1 and 3), and 0.93 (raters 1 and 3).

**Table 1.** Lin's concordance correlation coefficient calculated for both variables of the Esophageal Lichen Planus Severity Score (inflammation and fibrostenosis) separately and together

	INFL first	INFL last	FIBRO first	FIBRO last	Total first	Total last
Rater 1 and 2	0.76	0.67	0.78	0.67	0.77	0.75
Rater 1 and 3	0.70	0.69	0.80	0.67	0.74	0.76
Rater 2 and 3	0.90	0.95	0.98	1.00	0.93	0.98

Concordance was determined for both the first and last esophagogastroduodenoscopy for all 22 patients.

FIBRO, fibrostenosis; INFL, inflammation.

### Statistical analysis

Descriptive statistics were used to summarize demographic/patient characteristic variables and summarized as rates for categorical variables and medians along with interquartile range for continuous variables. The difference in continuous outcomes across compared groups was assessed using the nonparametric Mann-Whitney *U* test and summarized as mean difference along with 95% confidence intervals (CIs). The difference in categorical outcomes across compared groups was assessed using χ^2^ or Fisher exact test and summarized as odds ratio along with 95% CI.

We performed a Kaplan-Meier time-to-event analysis where we assessed the difference in response rates over the follow-up period for patients treated with TAC compared with patients not treated with TAC. The difference was assessed using the log-rank test. To assess the change in frequency of dilations between the groups, we created a ratio of the number of dilations over the total follow-up time.

The statistical significance for all comparisons is set at 5%. All statistical analysis will be completed using SPSS 25.0 ESD for Macintosh.

## RESULTS

### Patient characteristics

**Figure 2.**
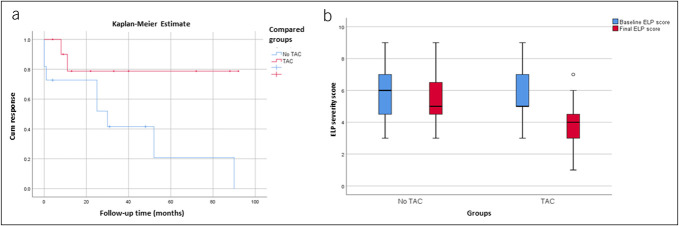
(**a**) Kaplan-Meier plot of response over time in patients undergoing tacrolimus treatment (TAC) vs no treatment; (**b**) box plot illustrating pre-post Esophageal Lichen Planus Severity Score in the TAC vs no-TAC group. The horizontal black lines denote median values, and the boxes extend from the 25th to the 75th percentile. The vertical extending lines represent adjacent values, and the dots represent the outliers. ELP, esophageal lichen planus.

Twenty-two patients underwent more than one EGD and were included in the study. Half (11) received tacrolimus, and half (11) received other therapy (i.e., cyclosporine, topical steroids, curcumin, or none; Table 2). Mean ELPSS on first EGD was similar between groups with 5.8 points (±1.8) for Tac and 5.9 points (±1.9) for No-Tac (*P* = 0.9). Among those on Tac vs No-Tac, there was a statistically significant decrease in the mean ELPSS to 3.6 and 5.6, respectively (mean difference 1.8 pts; 95% CI 0.25–3.38; *P* = 0.02) (Figure 2b).

**Table 2.** Baseline patient and treatment characteristics

Baseline and treatment data		
	Tacrolimus (n = 11)	No tacrolimus (n = 11)
Age	63	73
BMI	26	24
Female gender	11 (100%)	8 (72%)
Oral LP	11 (100%)	9 (82%)
Anogenital LP	7 (63%)	3 (27%)
Skin LP	4 (36%)	1 (9%)
Dysphagia	11 (100%)	11 (100%)
Odynophagia	2 (18%)	0
Weight loss	3 (27%)	3 (27%)
Esophageal Sx first	4 (36%)	9 (82%)
Follow-up	693 d	771 d
Medications/doses	1 mg b.i.d. (8) 2 mg b.i.d. (3)	Cyclosporine (2) Topical steroids (3) Curcumin (1) None (8)
Use of multiple therapies	2 (18%)	3 (27%)

BMI, body mass index; LP, lichen planus; Sx, symptoms.

There were no statistically significant differences in baseline characteristics between the groups (Tac and No-Tac), as shown in Table 2. In the Tac group, median age was 63, BMI 26, and 100% were female. The No-Tac group had median age of 73, BMI of 24, and 72% female. Extraesophageal manifestations in Tac and No-Tac were oral (100% vs 82%), anogenital (63% vs 27%), and skin (36% vs 9%). Most common symptoms were dysphagia (100% in both groups) followed by weight loss (27% in both groups). Median follow-up time was similar between the groups at 693 days for Tac compared with 771 days for No-Tac.

### Outcomes

The response rate in the Tac arm was 89% (9/11) vs 30% (3/11) in the No-Tac arm (*P* = 0.04) (Figure 2a).

In total, 108 successful dilations were performed across both groups. All dilations were performed with bougie dilators. Of the 22 patients, 12 had Savary dilation, 2 had Maloney, and 8 had both. The max diameter achieved was 16 mm in both groups. Comparing the last dilation with the first, patients in the Tac group ended with a slightly higher diameter (difference of 2.2 mm vs 1.5 mm); however, this was not significant. There were 2 minor complications—one patient in the Tac group had self-limiting bleeding, and one patient in the No-Tac group had mild discomfort after dilation that did not require treatment.

Assessment of the change in frequency of dilations between the groups according to the ratio of the number of dilations over the total follow-up time showed that patients on Tac required less total dilations over time compared with No-Tac, although this difference was not statistically significant (*P* = 0.19; mean difference 0.02; 95% CI −0.01 to 0.05).

Two patients in the Tac group stopped the medication because of (i) hyperglycemia and (ii) a mild rise in creatinine.

## DISCUSSION

The diagnosis and management of ELP are challenging, given the lack of validated diagnostic criteria and consensus on optimal management. This is largely due to the rarity of the disease and unfamiliarity among gastroenterologists, who are likely to encounter these patients first.

A scoring system was developed by Schauer et al, which uses histologic, direct immunofluorescence, and endoscopic findings together (17). Although this would be ideal, it may be unrealistic because of the significant variability amongst pathologists and the relatively nonspecific nature of the pathologic findings themselves. For the community gastroenterologist who is largely a visual learner and diagnostician, an easy-to-use scoring system with solely visual components will be more realistically used. It will also be easier when comparing their overall improvement on subsequent endoscopies. A similar visual scoring system is used in EoE—the EoE Reference Score, which assesses the severity of the most common findings seen: edema, rings, exudates, furrows, and strictures. Similarly, the ELPSS assesses the most common findings of the disease—pale/edematous mucosa, thick white exudate, sloughing or peeling, and degree of stricture formation. We find that the inter-rater variability is acceptable with our scoring system; however, a larger study with greater power will be needed to determine its validity.

Once the diagnosis is made, often in conjunction with a dermatologist as these patients commonly have concurrent oral or skin disease, a decision about therapy is the next challenge. Oral steroids are strongly advised against given their well-known complications and side effects. A variety of anti-inflammatory or immunosuppressant agents have been described in an extremely small number of patients and not repeated in literature likely because of their lack of follow-up and data. As topical steroids have become a cornerstone of therapy for EoE, another fibrostenotic inflammatory disorder of the esophagus, it follows suit that they were used in ELP. There are data suggesting patients will respond to topical steroid therapy, although this may be a more effective treatment in milder forms of disease. We suspect that patients with more moderate to severe disease will be better served by stronger medication, as was seen at our institution.

To the best of our knowledge, oral tablet tacrolimus has not been used in ELP. Tacrolimus, a calcineurin inhibitor that can reduce T-cell proliferation, has been used in topical form effectively for oral LP and commonly used as an alternative to topical steroids in skin disease. Tablet tacrolimus' use in allograft transplantation is well established with doses of 0.1–0.2 mg/kg/d, which can amount to 8–15 mg/d for an average adult. At our institution, we achieved a clinical response in ELP patients with oral tablet tacrolimus at doses up to 1–2 mg twice daily (a most of 4 mg daily). Thus, our ELP doses are 50%–75% less than transplant doses, which also suggests a proportional decrease in side effects, including neuro/nephrotoxicity, hypertension, or gastrointestinal complaints.

In our study, oral tablet-form tacrolimus in low doses (1–2 mg twice daily) significantly reduced the inflammatory component of ELP as evidenced by a significant reduction in the ELPSS between the first and final EGDs from 5.8 to 3.6 compared with a minimal reduction of 5.9–5.6 in patients on other therapy. This, in turn, likely reduced the degree of fibrostenosis as evidenced by the need for less frequent bougie dilations. At this low dose, only 2 patients stopped treatment, one because of hyperglycemia and the other a mild rise in creatinine, neither of which we could attribute directly to the use of tacrolimus. The other available treatments, as highlighted in our review article, are oral steroids (not preferred because of their innumerable systemic side effects), retinoids (ineffective in most), other various drugs (adrenocorticotropic hormone, rituximab, adalimumab, and cyclosporine) each reported in 1–2 patients only, and topical steroids (viable option in patients with milder disease) (6). Thus, we see that low-dose tacrolimus has a preferred safety profile, is effective, and can be used long term, providing lasting relief to this subset of patients with moderate to severe ELP.

There are limitations in our study, namely the small number of patients and our scoring system, which is not validated. However, given the retrospective nature of our study, a blinded assessment is not possible, and these results provide hypothesis to be pursued in future studies. Another possible limitation could be the lack of histologic response. However, the histological findings of LP tend to be nonspecific except for Civatte bodies, which explains some of the trouble making the original diagnosis. For this key reason, we did not do follow-up biopsies. We hope that both our scoring system and oral tablet tacrolimus, which proved useful for our patients, can be used by others, and inspire further research in this disease, which is less rare than we once thought.

## CONFLICTS OF INTEREST

**Guarantor of the article:** Joel E. Richter, MD.

**Specific author contributions:** All authors planned and drafted the manuscript. All authors have approved the final draft.

**Financial support:** None to report.

**Potential competing interests:** None to report.
